# TRPA1 and Sympathetic Activation Contribute to Increased Risk of Triggered Cardiac Arrhythmias in Hypertensive Rats Exposed to Diesel Exhaust

**DOI:** 10.1289/ehp.1003200

**Published:** 2011-03-04

**Authors:** Mehdi S. Hazari, Najwa Haykal-Coates, Darrell W. Winsett, Q. Todd Krantz, Charly King, Daniel L. Costa, Aimen K. Farraj

**Affiliations:** 1 Environmental Public Health Division, and; 2 Office of Research and Development, U.S. Environmental Protection Agency, Research Triangle Park, North Carolina, USA

**Keywords:** air pollution, arrhythmia, cardiac, diesel exhaust, sympathetic, TRPA1

## Abstract

Background: Diesel exhaust (DE), which is emitted from on- and off-road sources, is a complex mixture of toxic gaseous and particulate components that leads to triggered adverse cardiovascular effects such as arrhythmias.

Objective: We hypothesized that increased risk of triggered arrhythmias 1 day after DE exposure is mediated by airway sensory nerves bearing transient receptor potential (TRP) channels [e.g., transient receptor potential cation channel, member A1 (TRPA1)] that, when activated by noxious chemicals, can cause a centrally mediated autonomic imbalance and heightened risk of arrhythmia.

Methods: Spontaneously hypertensive rats implanted with radiotelemeters were whole-body exposed to either 500 μg/m^3^ (high) or 150 μg/m^3^ (low) whole DE (wDE) or filtered DE (fDE), or to filtered air (controls), for 4 hr. Arrhythmogenesis was assessed 24 hr later by continuous intravenous infusion of aconitine, an arrhythmogenic drug, while heart rate (HR) and electrocardiogram (ECG) were monitored.

Results: Rats exposed to wDE or fDE had slightly higher HRs and increased low-frequency:high-frequency ratios (sympathetic modulation) than did controls; ECG showed prolonged ventricular depolarization and shortened repolarization periods. Rats exposed to wDE developed arrhythmia at lower doses of aconitine than did controls; the dose was even lower in rats exposed to fDE. Pretreatment of low wDE–exposed rats with a TRPA1 antagonist or sympathetic blockade prevented the heightened sensitivity to arrhythmia.

Conclusions: These findings suggest that a single exposure to DE increases the sensitivity of the heart to triggered arrhythmias. The gaseous components appear to play an important role in the proarrhythmic response, which may be mediated by activation of TRPA1, and subsequent sympathetic modulation. As such, toxic inhalants may partly exhibit their toxicity by lowering the threshold for secondary triggers, complicating assessment of their risk.

Whole diesel exhaust (wDE) has been identified as a significant hazard to human health in a 2003 Integrated Risk Information System assessment by the U.S. Environmental Protection Agency (EPA 2003). Acute exposures to wDE elicit a spectrum of effects on the respiratory tract that include inflammation and congestion, as well as physiological symptoms such as coughing and shortness of breath (Ris 2007). The concern over these multiple respiratory effects coincides with a growing awareness that wDE exposure causes adverse cardiac events as well, particularly in people who have underlying diseases such hypertension and heart disease. Recently, a study by Mills et al. (2007) indicated that wDE could affect cardiac function in humans with preexisting heart disease. However, only a few epidemiological studies have reported that a single exposure to diesel-dominated traffic can compromise the electrical function and compensatory mechanisms of the heart, particularly in the 24 hr immediately after exposure. Among them are reports of increased incidence of cardiac arrhythmias immediately after exposure to air pollution (Link and Dockery 2010; Peters et al. 2000), including DE (Anselme et al. 2007). These findings have stirred great interest in these potentially serious outcomes, but much is still unknown about the mechanisms underlying them.

We previously demonstrated that in normal rats a single exposure to particulate matter (PM) or gaseous air pollutants has the potential to “sensitize” the heart to subsequent arrhythmogenic stimuli, which is further worsened by the presence of underlying cardiovascular disease (Hazari et al. 2009). Furthermore, it has become clear that underlying cardiovascular disease plays an important role in the body’s ability, or inability, to maintain cardiac homeostasis in the presence of stressful stimuli hours to days after exposure. The American Heart Association statement on PM air pollution and cardiovascular disease proposed several biological pathways by which air pollution, PM in particular, can produce such a compromised state and affect cardiovascular disease (Brook et al. 2010). Of the known mechanisms, acute exposure-related increase in “arrhythmia potential” is often linked to autonomic nervous system reflex arcs, which are putatively initiated when sensory irritant nerves are activated in the nose and lungs, and which result in brainstem-derived changes in sympathetic and parasympathetic balance thereafter. The airways are innervated by sensory nerves bearing transient receptor potential (TRP) channels, namely, member A1 (TRPA1) and member V1 (TRPV1), which detect different types of noxious chemicals, including many of those found in the complex mixtures of common air pollutants such as DE. Activation of these nerves by airborne irritants such as ozone or acrolein causes centrally mediated autonomic “imbalance,” which produces ventilatory, pulmonary, and cardiovascular function changes (Bautista et al. 2006; Bessac and Jordt 2008; Ghelfi et al. 2008).

In this study, we used aconitine, which targets voltage-dependent sodium channels in the myocardium, suppresses their inactivation, and thereby interferes with repolarization of the cardiomyocyte membrane in preparation for the next beat, as a challenge test (Hazari et al. 2009) to measure the arrhythmia sensitivity of spontaneously hypertensive (SH) rats after a single exposure to wDE. The aim of these experiments was to examine the role of TRP channels in the heightened arrhythmia sensitivity after wDE exposure and to determine if autonomic changes mediate the response. We hypothesized that a single exposure to wDE would sensitize the heart to aconitine-induced arrhythmia, a response that could be prevented by blocking TRPA1 (and possibly TRPV1) or by sympathetic modulation. It is believed that blockade of this receptor would prevent activation of airway sensory nerves and the autonomic reflex arc and would thus prevent the heightened response to aconitine by severing the outgoing signal that connects the autonomic nervous system to the heart.

## Materials and Methods

*Animals.* Male spontaneously hypertensive (SH) rats 18–20 weeks of age weighing 300–400 g were obtained from Charles River Laboratories (Wilmington, MA). Upon arrival, animals were housed two per cage with food and water available *ad libitum* in a facility approved by the Association for Assessment and Accreditation of Laboratory Animal Care International; all animals were treated humanely and with regard to alleviation of suffering. All experimental protocols were approved by and in accordance with the guidelines of the Institutional Animal Care and Use Committee of the U.S. EPA.

*Radiotelemeters, and electrocardiograms (ECG).* Methods are described in detail in Supplemental Material, Section IA (doi:10.1289/ehp.1003200). In brief, radiotelemeters were implanted in all animals as previously described (Hazari et al. 2009); this methodology tracks changes in cardiovascular function by monitoring ECG and heart rate (HR). Each animal was aseptically implanted with a radiotelemetry transmitter (model TA11CTA-F40; Data Sciences International, St. Paul, MN) in the abdominal cavity, and electrode leads were guided and secured in positions that approximated those of the lead II of a standard ECG. Using a remote receiver (DataART2.1; Data Sciences International, Inc.), HR and ECG waveforms were continuously acquired and saved during the 5-min baseline period and aconitine challenge.

We used ECGAuto software (EMKA Technologies USA, Falls Church, VA) to visualize individual ECG signals, analyze and quantify ECG segment durations, calculate time-domain and frequency-domain measures of heart rate variability (HRV), and identify cardiac arrhythmias. The Lambeth conventions (Walker et al. 1988) were used as guidelines for the identification of cardiac arrhythmic events in rats. Arrhythmias were identified as occurring sequentially during aconitine challenge as ventricular premature beats (VPBs), ventricular tachycardia (VT), and ventricular fibrillation (VF) [see Supplemental Material, Figure 1 (doi:10.1289/ehp.1003200)].

**Figure 1:**
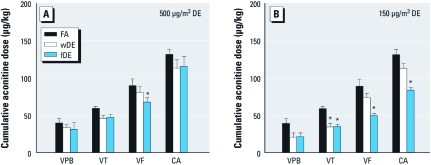
A single exposure to DE increases aconitine-triggered arrhythmia in hypertensive rats. Constant infusion of aconitine (2 μg/min) triggers VPB, followed by VT, VF, and progression to cardiac arrest (CA) in FA-exposed SH rats. The cumulative dose of aconitine necessary to trigger arrhythmia and CA in SH rats exposed to high wDE or fDE (*A*) or low wDE or fDE (*B*) was lower than for FA-exposed rats. Values are mean ± SE; *n* = 5–6. *p < 0.05 compared with FA controls.

*Groups.* Table 1 shows the experimental groups. SH rats (*n* = 6/group) were assigned to a group within one of three experiments:

**Table 1:** Experimental groups.

Table 1. Experimental groups.	
Experiment/exposure	Treatment
wDE vs. fDE	
FA	
High wDE	
High fDE	
Low wDE	
Low fDE	
wDE and TRP	
Low wDE	Vehicle
	TRPA1 antagonist (5 mg/kg, IP)
	TRP antagonist (2.5 mg/kg, IP)
	TRPV1 antagonist (5 mg/kg, IP)
FA	TRPA1 antagonist (5 mg/kg, IP)
	TRP antagonist (2.5 mg/kg, IP)
	TRPV1 antagonist (5 mg/kg, IP)
wDE and autonomics	
Low wDE	Vehicle
	Vagotomy
	Atropine (0.5 mg/kg, IP)
	Guanethidine (5 mg/kg, IP)
FA	Vagotomy
	Atropine (0.5 mg/kg, IP)
	Guanethidine (5 mg/kg, IP)
IP, intraperitoneal; TRPA1 antagonist, HC030031; TRP antagonist, RR; TRPV1 antagonist, SB366791.

- wDE versus filtered DE. These experiments were conducted to determine if lowering the concentrations of wDE would decrease arrhythmia risk and to assess the impact of only the gaseous components of wDE.
- DE and TRP. Animals were pretreated [before exposure to either filtered air (FA) or wDE] with a TRPA1 antagonist (HC030031), a general TRP antagonist [ruthenium red (RR)], or a TRPV1 antagonist (SB366791). These experiments were conducted to assess the role of TRP channels, which are located on the irritant sensory nerves of the upper and lower airways, on arrhythmia sensitivity after exposure to low wDE (150 μg/m^3^ for 4 hr).
- DE and autonomics. Animals received bilateral vagotomy, the sympathetic adrenergic blocker guanethidine, or the muscarinic (parasympathetic) antagonist atropine after exposure to either FA or wDE (30 min before aconitine challenge). These experiments were conducted to examine the role of the parasympathetic and sympathetic branches, which regulate heart function, in the arrhythmia response after exposure to low wDE (150 μg/m^3^ for 4 hr).

*DE generation and exposure.* The method for generation of wDE has been previously described (Sharkhuu et al. 2010) [see also Supplemental Material, Section IB (doi:10.1289/ehp.1003200)]. Briefly, wDE for exposure experiments was generated using a Yanmar diesel generator using low-sulfur diesel fuel (32 ppm). From the engine, the exhaust was mixed with particulate (HEPA)-filtered room air (FA). wDE concentrations were based on the fine PM fractions of the diluted exhaust (mass median aerodynamic diameter < 2.5 µm; PM_2.5_). Target concentrations were 500 µg PM/m^3^ (high) and 150 μg PM/m^3^ (low), which were routed to a filtered and unfiltered exposure chamber. The filtered chamber had almost no PM present but contained all the diluted combustion gases present in the unfiltered chamber [filtered DE (fDE)]. Control animals were placed in a third chamber supplied with FA. Continuous emission monitors were used to measure chamber concentrations of PM, oxygen, carbon monoxide (CO), nitrogen oxides (NO_x_), and sulfur dioxide (SO_2_) [see Supplemental Material, Table 1 (doi:10.1289/ehp.1003200)]. Chamber temperatures, relative humidity, and noise were also monitored and maintained within acceptable ranges.

*Aconitine challenge.* Twenty-four hours after exposure, all animals were anesthetized with intraperitoneal (IP) urethane (1.5 g/kg) and underwent the aconitine challenge; supplemental doses of the anesthetic were administered intravenously when necessary to abolish pain reflex. Animal body temperature was maintained at approximately 36°C with a heating pad. The left jugular vein was cannulated with PE 50 (i.d., 0.58 mm; o.d., 0.965 mm) polyethylene tubing for the administration of aconitine. Aconitine (10 μg/mL) was continuously infused at a speed of 0.2 mL/min while ECG was continuously monitored and timed. Sensitivity to arrhythmia was measured as the threshold dose of aconitine required to produce VPBs, VT, and VF, calculated as 10 μg/mL × 0.2 mL/min × time required for inducing arrhythmia (minutes)/body weight (kilograms).

*Drugs.* The specific TRPA1 antagonist HC030031 (Chembridge, San Diego, CA) was dissolved in dimethyl sulfoxide (DMSO). The general TRP antagonist RR (Sigma-Aldrich, St. Louis, MO) and the specific TRPV1 antagonist SB366791 (Tocris, Ellisville, MO) were dissolved in 50% DMSO and 50% saline. Guanethidine monosulfate (U.S. Pharmacopeia, Rockville, MD) and atropine (Sigma-Aldrich) were dissolved in saline. Stock solutions of aconitine (Sigma-Aldrich) were dissolved in ethanol and then diluted to the desired concentration with saline.

*Statistics.* Statistical analyses for all data in this study were performed using SAS software (version 9.1.3; SAS Institute Inc., Cary, NC). PROC MIXED and PROC GLIMMIX procedures were used to analyze all ECG- and HRV-generated data. We performed tests of normality for all continuous variables and used parametric methods of analysis. A linear mixed model with restricted maximum-likelihood estimation analysis (SAS) and least squares means post hoc test were used to determine statistical differences for all data. All aconitine dose–response data were analyzed using an analysis of variance for repeated measures; *p* < 0.05 was considered statistically significant. Reported values represent means ± SE.

## Results

*Heart rate.* Twenty-four hours after exposure, urethane-anesthetized SH rats exposed to high or low wDE or fDE had slightly higher HRs than did FA controls [see Supplemental Material, Figure 2 (doi:10.1289/ehp.1003200)]. HR did not increase in response to low wDE in rats pretreated with the TRPA1 antagonist, but rats pretreated with either the TRP antagonist or TRPV1 antagonist had a larger (nonsignificant) increase in HR after wDE than did untreated rats. HR increased significantly after low wDE exposure (*p* < 0.05 vs. FA controls) in vagotomized rats and rats treated with atropine, but the increase in HR was less pronounced in rats treated with guanethidine.

**Figure 2:**
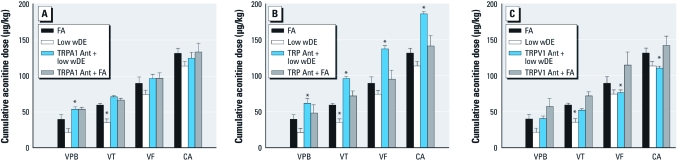
Heightened arrhythmia sensitivity after DE exposure is mediated by TRPA1. Constant infusion of aconitine (2 μg/min) triggers VPB, followed by VT, VF, and progression to cardiac arrest (CA) in FA-exposed SH rats. The cumulative dose of aconitine necessary to trigger arrhythmia and CA in SH rats exposed to low wDE was lower than for those exposed to FA; (*A*) this response was blocked by the TRPA1 antagonist (Ant; HC030031; *A*). The TRP antagonist RR caused a significant decrease in sensitivity relative to FA (*B*), but the TRPV1 antagonist SB366791 only reduced sensitivity to VPB and VT (*C*). The drugs had no effect on controls. Values are mean ± SE. *p < 0.05 compared with the corresponding FA control.

*ECG.* After exposure to wDE or fDE, we observed a prolongation of ventricular depolarization and a shortening of ventricular repolarization, indicated by an increase in QRS duration and decrease in ST segment duration, respectively [see Supplemental Material, Figure 3 (doi:10.1289/ehp.1003200)]. We saw no indication of heterogeneity of repolarization, as indicated by QTc. Pretreatment of rats with the TRPA1 antagonist, TRP antagonist, or TRPV1 antagonist prevented the increase in QRS duration, and in fact, pretreatment with the TRPA1 or TRP antagonist actually caused a significant increase in the ST segment duration. Vagotomy and atropine appeared to prevent the increase in QRS duration caused by low wDE but not the decrease in ST segment duration. Postexposure guanethidine treatment seemed to partially reverse effects of wDE on ST segment duration but did not appear to alter QRS duration or QTc (corrected QT interval) in response to exposure. We observed no significant effects of the drugs on FA rats (data not shown).

**Figure 3:**
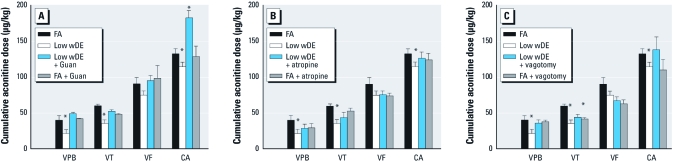
Heightened arrhythmia sensitivity after DE exposure is mediated by sympathetic activation. Constant infusion of aconitine (2 μg/min) triggers VPB, followed by VT, VF, and progression to cardiac arrest (CA) in FA-exposed SH rats. The increased arrhythmia response to aconitine after low wDE was prevented by acute sympathetic blockade with guanethidine (Guan; *A*) but not by muscarinic blockade with atropine (*B*). Vagotomy only partially reduced the low wDE–induced proarrhythmic response (*C*). Sympathetic blockade had no effect in FA-exposed animals (*A*); however, muscarinic blockade (*B*) and vagotomy (*C*) increased aconitine-induced arrhythmia sensitivity in FA rats. Values are mean ± SE. *p < 0.05 compared with the corresponding FA control.

*Heart rate variability.* Rats exposed to wDE or fDE had nonsignificant decreases in R-R intervals compared with FA controls [see Supplemental Material, Table 2 (doi:10.1289/ehp.1003200)]. When adjusted for HR, only rats exposed to low wDE and low fDE had increases in the time-domain HRV measures [standard deviation of the time between normal-to-normal beats (SDNN) and root mean squared successive differences (RMSSD)] compared with controls. The low frequency/high frequency (LF/HF) ratio, an estimate of the relative balance between sympathetic (LF) and vagal (parasympathetic, HF) activity, was increased in all DE-exposed rats compared with controls, but the difference was significant for high fDE only. Treatment of rats with the TRPA1 antagonist appeared to prevent effects of low wDE on R-R, SDNN, RMSSD, and the LF/HF ratio. The decline in R-R was more pronounced after low wDE exposure in rats pretreated with TRP and TRPV1 antagonists than in rats that were not pretreated. SDNN, RMSSD, and LF/HF ratios increased after low wDE exposure in rats without pretreatment but decreased after exposure in rats pretreated with TRP or TRPV1 antagonists. As expected, vagotomy decreased RR, SDNN, and RMSSD and increased LF/HF ratio in FA rats. In low wDE–exposed rats, vagotomy caused similar but even more pronounced changes in these parameters, which were significantly different from untreated low wDE rats. Atropine and guanethidine had little effect in FA rats. However, atropine and guanethidine decreased all parameters, some significantly, with respect to untreated low wDE–exposed rats.

*Aconitine challenge.* During aconitine infusion, the first arrhythmia to manifest was the VPB; continued infusion of aconitine then elicited three or more successive VPBs or VT, and then VF. Figure 1A shows the dose of aconitine that elicited arrhythmia in SH rats exposed to high wDE. The cumulative dose of aconitine necessary to trigger VPB, VT, and VF was lower in rats exposed to high wDE than in controls. High fDE appeared to be as potent as high wDE, or more so in the case of VF. The cumulative dose of aconitine needed to trigger the same arrhythmias was lower, but significantly so only for VT, in rats exposed to low wDE than in FA rats, and like the high fDE, low fDE was either as potent as low wDE in sensitizing animals to arrhythmogenesis or more potent (Figure 1B).

Figure 2 shows the responses to aconitine in the low wDE and TRP experiments. Rats pretreated with the TRPA1 antagonist HC030031 did not show increased responsiveness to aconitine after low wDE exposure (Figure 2A), whereas pretreatment with the general TRP antagonist RR appeared to make the animals hyporesponsive to aconitine after low wDE (Figure 2B). The TRPV1 antagonist SB366791 appeared to prevent increased sensitivity to aconitine caused by low wDE based on VPB and VT, but aconitine doses required to elicit VF and cardiac arrest after wDE were comparable to those in rats without pretreatment (Figure 2C). None of the drugs influenced responsiveness to aconitine in the FA rats.

Treatment with the sympathetic blocker guanethidine before aconitine challenge prevented the potentiated arrhythmic response to aconitine and significantly increased the dose necessary to cause cardiac arrest in low wDE–exposed rats; it had no effect on controls (Figure 3A). On the other hand, the muscarinic antagonist atropine did not affect sensitivity to aconitine after low wDE, and vagotomy prevented it for only VPB. More significant, FA rats vagotomized or treated with atropine developed arrhythmia at lower cumulative doses of aconitine than did vehicle-treated controls; these responses resembled those of low wDE–exposed rats (Figure 3B,C).

## Discussion

The data presented here suggest that a single exposure to a complex air pollutant increases susceptibility to triggered cardiac arrhythmias 1 day after exposure. Although we expected the degree to which high wDE increased arrhythmia sensitivity, the comparable potency of low wDE was surprising. These experiments also suggest that the chemosensor TRPA1, which is stimulated by irritant gases (e.g., acrolein) typically present in wDE (Krivoshto et al. 2008), likely contributes to the proarrhythmic response by causing autonomic imbalance and a shift toward sympathetic activation. These results, considered with the strong effect of fDE (gases alone), point to the complexity of studying the short-term cardiac effects of multipollutant mixtures and the interaction of their components at certain concentrations.

Few studies have demonstrated that a single air pollution episode directly alters cardiac electrophysiology in a manner that would be potentially detrimental. However, as we report here, the effect of one exposure on heart rhythm may be latent and indirect, altering the degree to which the cardiovascular system can withstand stress and/or lowering the threshold for initiation of adverse ventricular arrhythmias in response to a stimulus, particularly in compromised individuals. Regarding wDE, Anselme et al. (2007) previously showed a 200–500% increase in VPB arrhythmias in chronic heart failure (CHF) Wistar rats exposed to 500 μg/m^3^ wDE, which persisted for > 5 hr, but they saw no change in wDE-exposed healthy rats. Therefore, the challenge approach employed in our study reveals a unique way to identify subtle impacts of air pollution exposure that may be effective not only in animals with underlying cardiovascular disease, such as the CHF Wistar rats or SH rats, but also, as we have previously shown (Hazari et al. 2009), in healthy strains such as WKY.

The finding that low wDE—which had PM concentrations comparable to those in human studies (Kipen et al. 2010; Mills et al. 2007, 2011)—caused arrhythmia sensitivity comparable to that caused by high wDE was unexpected. A search of the relevant literature indicates that this lack of dose dependency has been reported by others. Hansen et al. (2007) found that low wDE exposure caused significant impairment of endothelium-dependent vasorelaxation in mildly atherosclerotic mice, whereas high wDE had no effect. Studies have shown that lower particle masses of DE contain a greater fraction of ultrafine and nanosized particles (Bagley et al. 1996; Johnson and Baumgard 1996), which could penetrate more distally and increase the degree of sensory activation, thus accounting for the effects observed with low wDE. Additionally, these smaller particles have a larger total surface area onto which known toxic organic compounds from DE, such as polycyclic aromatic hydrocarbons (PAHs), can adsorb and ultimately be transported deeper into the lung (Ris 2007). Thus, it is likely that chemical composition, as demonstrated with concentrated ambient particles (CAPs) with higher metals and organic carbon concentrations (Kodavanti et al. 2005), may be a more important determinant of biological effects than shear particle mass. To be clear, however, our results do not suggest that higher wDE concentrations are less toxic; rather, it may be that the toxicity profile differs for high and low concentrations. Saito et al. (2002) observed similar inversed responses with wDE exposures in mice, where 100 μg/m^3^ wDE caused an increase in interleukin-4 in mouse lungs, which they suggested contributed to development of allergic airways, whereas 3 mg/m^3^ wDE resulted in suppression.

wDE comprises not only PAHs but also highly toxic gases, which include NO_x_, sulfur oxides, ozone, CO, and various aldehydes (e.g., acrolein) (Krivoshto et al. 2008; Ris 2007). Although many studies and assessments stress the adverse effects of PM (Metzger et al. 2007; Wellenius et al. 2002), our findings suggest an important role for the gases, the concentrations of which were not affected by particle removal, in promoting cardiac arrhythmogenesis in the short term (Hesterberg et al. 2009). It may be that the presence of particles changes the composition, and therefore toxicity, of the gaseous components of wDE because of adsorption or chemical transformation. Regardless, several epidemiological studies have shown a significant association between increases in ventricular arrhythmias and increased levels of nitrogen dioxide and CO (Peters et al. 2000) and SO_2_ (Ljungman et al. 2008; Rich et al. 2006). Most of these gases are considered to be serious respiratory irritants, yet information on the short-term cardiovascular consequences of exposure is limited, particularly with respect to reflex control. Levels of CO and NO_x_ were significantly higher in our wDE exposures compared with FA. However, although the level of these gases was higher in high wDE than in low wDE, the latter caused greater sensitivity to arrhythmia, suggesting the response may be driven more by other components such as aldehydes, which were not measured during exposure but should not be ruled out given their irritant characteristics (Hazari et al. 2008).

**Figure 4:**
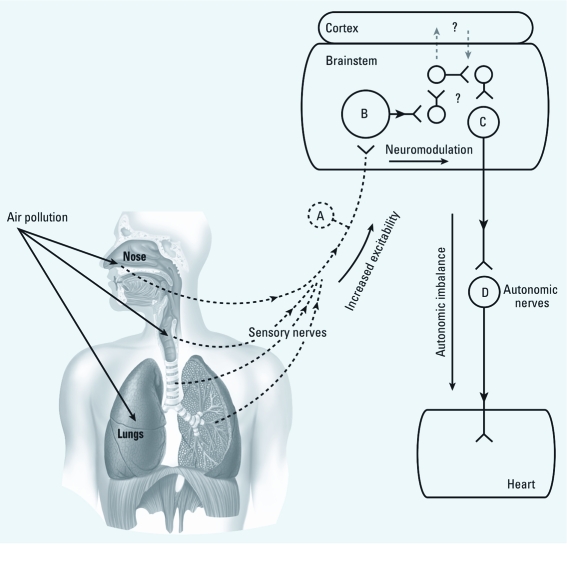
Simplified depiction of how exposure to air pollutants is proposed to sensitize the sensory-to-autonomic reflex arc and alter subsequent responses. Activation of airway sensory nerves (*A*) causes local effects and stimulates neurons in the midbrain (*B*). Neural circuits within the midbrain process the signals and then activate the preganglionic autonomic neuron (*C*); postganglionic autonomic neurons (*D*) carry the signal to the heart. Air pollution may increase the excitability of neuron A (sensitization), which would potentiate transmission in neuron B and, in turn, increase/decrease central nervous system outflow by neuron C thereafter (autonomic imbalance). Adapted from Undem et al. (2000).

Exposure to inhaled irritants (Nishino et al. 1996), including air pollution, has repeatedly been shown to cause immediate cardiac changes such as decreased HR, alterations in HRV and ECG, and dysrhythmia in humans (Gold et al. 2000; Pope et al. 1999). These reflexive effects are also seen in animals. However, animal studies have also demonstrated that significant activation of these sensory receptors causes a sensitization or priming of the reflex (Widdicombe and Lee 2001). For instance, ozone not only causes acute irritant responses through the airway sensory receptors but also sensitizes and enhances their excitability to subsequent stimuli (Ho et al. 1998; Joad et al. 1998). Similarly, exposure to sidestream tobacco smoke not only sensitized bronchopulmonary C-fibers but also transmitted this enhanced excitability to the nucleus tractus solitarius, which regulates outgoing autonomic signals, as well as other efferent information, to the heart and lungs (Mutoh et al. 2000). Thus, based on these data, we assumed that exposure to wDE would not only cause immediate exposure-related physiological changes (not measured) but also sensitize the airway autonomic reflex arc (Figure 4), resulting in autonomic imbalance and increased sensitivity to developing arrhythmia.

In the present study, we indirectly examined the role of airway C-fibers in increasing the sensitivity of animals to aconitine-induced arrhythmia after exposure to wDE by testing the involvement of TRPA1 and TRPV1 ion channels, which are the chemical-sensing structures colocalized to these fibers. TRPA1 is activated by irritants commonly found in wDE, such as acrolein (Bautista et al. 2006), ozone (Taylor-Clark and Undem 2010), oxidizing agents, and other aldehydes, whereas TRPV1 is activated by capsaicin, the pungent chemical found in red chilies. It was not entirely a surprise that TRPA1 antagonism prevented the wDE-induced heightened sensitivity to arrhythmia. Multiple studies have shown that an initial challenge may activate TRPA1 channels and, in the presence of inflammation, result in hypersensitivity to a host of other chemical stimuli thereafter (reviewed by Bessac and Jordt 2008). So, we assumed that blockade of TRPA1 not only inhibited sensitization of the afferent pathways but also prevented any acute or short-term changes in centrally mediated autonomic imbalance. The partial effects of TRPV1 may be explained by the fact that it can be activated secondary to TRPA1-mediated Ca^2+^ mobilization or through the release of inflammatory mediators (Bessac and Jordt 2008). As such, a role for TRPV1-mediated lung reflexes in triggering cardiac rhythm disturbances and oxidative stress after CAPs has already been shown (Ghelfi et al. 2008). Finally, our future experiments will seek to determine if TRPA1 mediates heightened sensitivity to arrhythmia in animals exposed to filtered DE; this would clarify whether the gases in wDE drive this response.

Air pollution has been shown to dysregulate the autonomic nervous system (reviewed by Simkhovich et al. 2008), linking exposure to both decreases (Gold et al. 2000) and increases (Pope et al. 1999) in HR. Most but not all studies have demonstrated a decrease in HRV, which has also been observed in animals exposed to residual oil fly ash (Wellenius et al. 2002) or CAPs (Godleski et al. 1997); these types of responses are consistent with increased sympathetic activity. Blockade of sympathetic activity using guanethidine, which targets peripheral adrenergic neurons, prevented heightened aconitine-induced arrhythmia, which seems to confirm the predominant finding of exposure-related enhancement of sympathetic tone in both humans and animals. It is yet unclear whether this protective effect is due to reduced cardiac rate/contractility or vasodilatation, both of which are the result of guanethidine treatment. Regardless, autonomic shift toward increased sympathetic drive after exposure to wDE predisposes the animal to triggered arrhythmia and, as such, represents a disruption of homeostatic balance one day after exposure. This is further illustrated by the fact that both atropine or vagotomy (removal of parasympathetic influence), which had minimal to no effect on aconitine sensitivity in response to wDE, increased arrhythmia sensitivity in rats exposed to air, suggesting that the balance of activity from these autonomic nerves is important in maintaining normal function both in a healthy and “challenged” state.

Paradoxically, time-domain HRV (SDNN and RMSSD) was decreased in rats exposed to high wDE or high fDE but increased in rats exposed to low wDE or low fDE. However, all DE-exposed rats had small increases in LF/HF ratios, except the significant increase observed with high fDE. Peretz et al. (2008) observed similar opposite HRV effects of high and low DE concentrations in healthy individuals and concluded that they did not observe a consistent effect on the autonomic control of the heart. Similarly, Anselme et al. (2007) found that HRV changes due to wDE exposure were only transient compared with persistent ventricular proarrhythmic effects, indicating that HRV may be only a marker of exposure and not directly involved in the mechanism of arrhythmogenesis. Instead, Anselme et al. (2007) suggested that the immediate proarrhythmic response was due to direct cardiac effects of certain wDE constituents that enter the circulation, whereas the persistent effect was due to inflammation and oxidative stress. Because we did not measure inflammation or oxidative stress, we cannot address their role in mediating these heightened arrhythmia responses. However, it is likely that these conditions alter the local tissue environment that controls the excitability of cardiac tissue and the neurons innervating it. Therefore, it remains to be determined whether the response of the autonomic nervous system is directly due to airway-mediated reflexes or secondary to inflammation and oxidative stress, or a combination of the two.

In the present study, blockade of TRPA1 prevented the increase in HRV induced by low wDE. We also observed these effects on HRV in vagotomized animals and those treated with atropine or guanethidine. However, in these data, the LF/HF ratio, which represents the balance between sympathetic and parasympathetic tone, may be the main indicator of protection against DE-induced arrhythmogenesis. Treatment with the TRPA1 antagonist or sympathetic blockade lowered the LF/HF ratio and prevented wDE-induced arrhythmia sensitivity. Additionally, the vagotomized FA-exposed rats developed increased arrhythmia sensitivity to aconitine and had higher LF/HF ratios, suggesting that autonomic imbalance marked by increased sympathetic drive may be mediating the proarrhythmic response.

We observed ECG changes 1 day after exposure, but it is unclear whether these changes would predispose to greater risk of developing arrhythmia. Exposure to wDE caused prolongation of the QRS duration, which represents lengthening of ventricular depolarization and is believed to predispose individuals with underlying heart disease to an increased risk of ventricular arrhythmias (Kashani and Barold 2005) and decreased ST segment length. Decreased ST segment length in response to wDE was prevented by both TRPA1 and sympathetic blockade, suggesting that the shorter duration of repolarization after wDE may also contribute to greater arrhythmia sensitivity. Similar ST segment decreases have been observed in human beings exposed to wDE (Mills et al. 2007). However, we found no evidence of repolarization abnormalities or ST depression.

## Conclusion

Traditional environmental health evaluations focus on the monotonic exposure–response paradigm, and although this approach is valuable, in the case of some physiological outcomes as might be observed in the cardiovascular system, such an approach does not take into account exposure-induced “sensitization” or “priming” of secondary responses from other triggers. The findings of this study demonstrate that a single exposure to wDE increases the sensitivity of the cardiac electrical conduction system (perhaps lowering a threshold) such that it increases the risk of triggered arrhythmias. How reversible these effects are remains to be determined. Additionally, evidence that fDE (i.e., gases alone) elicits more cardiotoxic effects at these exposure levels than does wDE suggests that source apportionment for multipollutant mixtures is more complex than simply attributing the effects to the sum total of individual components. It also appears that heightened arrhythmia sensitivity may be mediated by activation of TRPA1 (on airway sensory nerves), which is particularly sensitive to inhaled irritants, and autonomic imbalance with a shift toward sympathetic activation. Taken together, this work highlights the importance of air pollution–induced irritation in the nose and lungs on the persistent short-term effects of a single exposure, particularly for people with underlying cardiovascular disease, and warrants further investigation.

## Supplemental Material

(800 KB) PDF
