# Supplementary material for: TRPA1 and Sympathetic Activation Contribute to Increased Risk of Triggered Cardiac Arrhythmias in Hypertensive Rats Exposed to Diesel Exhaust
Source: Environ Health Perspect. 2011 Mar 4;119(7):951–7. doi: 10.1289/ehp.1003200 (PMC3223009; doi:10.1289/ehp.1003200)
Supplement: (800 KB) PDF [file ehp.1003200.s001.pdf]

SUPPLEMENTAL MATERIAL

TRPA1 and Sympathetic Activation Contribute to Increased Risk of Triggered Cardiac Arrhythmias in Hypertensive Rats Exposed to Diesel Exhaust

Mehdi S. Hazari[1], Najwa Haykal-Coates[1], Darrell W. Winsett[1], Q. Todd Krantz[1], Charly King[1], Daniel L. Costa[2] and Aimen K. Farraj[1]

[1]Environmental Public Health Division/[2]Office of Research and Development, United States Environmental Protection Agency, Research Triangle Park, NC, 27711

Disclaimer: This paper has been reviewed and approved for release by the National Health and Environmental Effects Research Laboratory, U.S. Environmental Protection Agency. Approval does not signify that the contents necessarily reflect the views and policies of the U.S. EPA, nor does mention of trade names.

Corresponding author: Mehdi S. Hazari, Environmental Public Health Division, USEPA, 109 Alexander Drive, B143-01; Research Triangle Park, NC 27711; (Phone: 919-541-4588; Fax: 919-541-0034; email: hazari.mehdi@epa.gov)

Running title: Role for TRPA1 in Diesel-Induced Arrhythmia Risk

TABLE OF CONTENTS

I. Materials and methods

A. Full description of *Implantation of radiotelemeters, and electrocardiogram acquisition and analysis*

B. Full description of *Diesel exhaust generation and exposure*

II. Tables

A. Supplemental Material, Table 1: Exposure Details

B. Supplemental Material, Table 2: Heart rate variability data

III. Figures

A. Supplemental Material, Figure 1. Typical electrocardiogram (ECG) traces showing normal sinus rhythm, ventricular premature beat (VPB), ventricular tachycardia (VT), and ventricular fibrillation (VF)

B. Supplemental Material, Figure 2. Diesel exhaust mediated increase in heart rate is only prevented by blocking TRPA1

C. Supplemental Material, Figure 3. Exposure to diesel exhaust causes a prolongation of ventricular depolarization and shortening of repolarization in the ECG

IV. References

I. Materials and methods:

A. *Implantation of radiotelemeters, and electrocardiogram acquisition and analysis.* Radiotelemeters were implanted in all animals as previously described (Hazari et al. 2009); this methodology was used to track changes in cardiovascular function by monitoring ECG and HR. Briefly, animals were weighed and anesthetized with ketamine hydrochloride/xylazine hydrochloride solution (1ml/kg, ip Sigma-Aldrich, St. Louis, MO). Using aseptic technique, each animal was implanted with a radiotelemetry transmitter (Model TA11CTA-F40, Data Sciences International, St. Paul, MN) in the abdominal cavity through a small incision. The electrode leads were guided through the abdominal musculature via separate stab wounds and tunneled subcutaneously across the lateral ventral thorax; the distal portions of the leads were secured in positions that approximated those of the lead II of a standard electrocardiogram (ECG). Body heat was maintained both during and immediately following the surgery. All animals were allowed 7-10 days to recover from the surgery and reestablish circadian rhythms. Using a remote receiver (DataART2.1: Data Sciences International, Inc., St. Paul, MN), ECG waveforms were continuously acquired and saved during the 5-min baseline period and aconitine challenge, which did not last longer than 40mins. HR was obtained from the ECG.

ECGAuto software (EMKA technologies USA, Falls Church, VA) was used to visualize individual ECG signals, analyze and quantify ECG segment durations and identify cardiac arrhythmias. Using ECGAuto, P wave, QRS complex, and T wave were identified for individual ECG waveforms and compiled into a library and used for analysis of all experimental ECG traces. The following parameters were determined for each ECG waveform: PR interval, QRS duration, QT corrected for HR (QTc) using Bazett's formula, and ST interval. The Lambeth

conventions (Walker et al. 1988) were used as guidelines for the identification of cardiac arrhythmic events in rats. Arrhythmias were identified as occurring sequentially during aconitine challenge as ventricular premature beats (VPBs), ventricular tachycardia (VT) and ventricular fibrillation (VF).

Heart rate variability (HRV) was also calculated as the mean of the differences between sequential HRs for the complete set of ECG signals. For each 1-min stream of ECG waveforms, mean time between successive QRS complex peaks (RR interval), mean HR, and mean HRV-analysis–generated time-domain measures were acquired. The time-domain measures included standard deviation of the time between normal-to-normal beats (SDNN), and root mean squared successive differences (RMSSD). HRV analysis was also conducted in the frequency domain using a fast-Fourier transform. In this study, the spectrum was divided into low-frequency (LF) and high-frequency (HF) regions. The ratio of these two frequency domains (LF/HF) was calculated as an estimate of the relative balance between sympathetic (LF) and vagal (HF) activity.

B. *Diesel exhaust generation and exposure.* wDE for exposure experiments was generated using a 4.8 kW (6.4 hp) direct injection single-cylinder 0.320 L displacement Yanmar L70 V diesel generator operated at a constant 3600 rpm. Resistance heating elements provided a constant 3 kW load. Low sulfur diesel fuel (32 ppm), purchased from a local distributor was available from a large storage tank. Engine lubrication oil (Shell Rotella, 15W-40) was changed before each set of exposure tests. From the engine, approximately 85 L/min of the exhaust was directed to a cone diluter and mixed with approximately 595 L/min (7:1 dilution) of high efficiency

particulate air (HEPA) filtered room air. The diluted exhaust then traveled approximately 12 m through 7.1 cm diameter stainless steel tubing to a Hazelton 1000 (984 L) exposure chamber housed in an isolated animal exposure room. Target wDE concentration of the diluted exhaust was 500 μg of particulate matter (PM)/m^3 (high) and 150 μg of PM/m^3 (low) which was routed to a filtered and unfiltered exposure chamber. Although the filtered chamber had nearly no PM present it still contained all the diluted combustion gases as the unfiltered chamber. The chamber concentrations were controlled by periodic adjustments of dilution air based on continuous mass concentrations determined by tapered element oscillating microbalance (TEOM, Rupprecht and Patashnick Co., series 1400, Albany, NY) instruments. These instruments include a heated (50 °C) chamber that could theoretically vaporize low temperature volatiles. Control animals were placed in a third chamber supplied with the same HEPA filtered room air. The chambers were operated at the same flow rate (424 L/min) which resulted in approximately 25 air exchanges per hour. Integrated 4 h filter samples (14.1 L/min) were collected daily from each chamber and analyzed gravimetrically to determine particle concentrations. Continuous emission monitors (CEMs) were used to measure chamber concentrations of PM by TEOM, oxygen (O_2, Beckman Corp., model 755, La Habra, CA), carbon monoxide (CO, Thermo Electron Corp., model 48, Franklin, MA), nitrogen oxides (NO and NO_2, Teledyne Technology Co., model 200A4, San Diego, CA), and sulfur dioxide (SO_2, Thermo Electron Corp, model 43c, Franklin, MA). Samples were extracted through fixed stainless steel probes in the exposure chambers. Gas samples were passed through a particulate filter prior to the individual gas analyzers. Dilution of air was adjusted periodically to maintain target PM concentrations as measured by the TEOM. Particle size distributions were characterized during each exposure using an engine exhaust

particle sizer (EEEPS, TSI Inc., model 3090, St. Paul, MN). Chamber temperatures, relative humidity, and noise were also monitored, and maintained within acceptable ranges.

II. Tables

A. Supplemental Material, Table 1. Exposure details

Exposure	PM conc ($\mu g/m^3$)	O_2 (%)	CO (ppm)	SO_2 (ppm)	NOx (ppm)	Temp (°F)
FA	0	21.0 ± 0.0	0.5 ± 0.2	0.0 ± 0.1	0.0 ± 0.0	73.1 ± 0.5
High wDE	512.4 ± 13.6	20.6 ± 0.0	10.6 ± 0.5*	0.2 ± 0.1	11.5 ± 0.8*	72.6 ± 0.7
High fDE	0	20.6 ± 0.0	10.4 ± 0.4*	0.4 ± 0.1	11.3 ± 1.2*	74.2 ± 0.4
Low wDE	153.4 ± 4.5	20.8 ± 0.0	4.2 ± 0.4*	0.0 ± 0.0	3.3 ± 0.6*	72.5 ± 0.7
Low fDE	0	20.8 ± 0.1	4.3 ± 0.6*	0.0 ± 0.0	3.8 ± 0.9*	72.0 ± 1.0

ppm = parts per million
Values are mean ± SEM
*significantly different from FA, $p < 0.05$
Note: the concentrations of other gases were not measured

B. Supplemental Material, Table 2. Heart Rate Variability

Experiment	Group	R-R (ms)	SDNN (ms)	RMSSD (ms)	LF/HF
I.	Air	276.5 ± 13.3	5.8 ± 0.7	8.1 ± 1.5	2.3 ± 0.6
	High wDE	248.1 ± 18.5	4.9 ± 0.5	5.8 ± 1.2	4.5 ± 2.0
	High fDE	228.7 ± 9.1	4.9 ± 1.1	6.1 ± 1.4	13.1 ± 7.5*
	Low wDE	267.8 ± 10.3	8.0 ± 1.6	10.4 ± 2.1	5.4 ± 1.0
	Low fDE	259.7 ± 10.6	15.3 ± 5.2*	23.8 ± 9.5*	3.3 ± 0.9
II.	Low wDE + HC030031	285.5 ± 11.7	5.1 ± 0.4	6.8 ± 0.7	1.8 ± 0.8
	Air + HC030031	272.1 ± 20.4	3.0 ± 0.9	3.7 ± 1.0	2.6 ± 1.9
	Low wDE + RR	237.9 ± 24.6	3.4 ± 0.8	4.2 ± 1.4	0.7 ± 0.1
	Air + RR	238.6 ± 12.8	3.8 ± 0.6	5.3 ± 1.3	1.7 ± 1.1
	Low wDE + SB366791	227.4 ± 14.1§	5.9 ± 1.5	5.5 ± 1.5	1.4 ± 0.4
	Air + SB366791	269.4 ± 0.2	6.1 ± 1.6	10.0 ± 3.0	2.0 ± 1.7
III.	Low wDE + vagotomy	161.4 ± 4.2§	1.4 ± 0.2§	1.1 ± 0.1§	9.8 ± 2.6§
	Air + vagotomy	186.3 ± 28.4	2.6 ± 1.9	3.4 ± 2.7	11.8 ± 3.1
	Low wDE + guanethidine	240.8 ± 13.6	3.9 ± 1.0	4.6 ± 1.1	1.1 ± 0.3
	Air + guanethidine	293.6 ± 10.1	6.1 ± 1.2	7.6 ± 1.9	2.6 ± 1.2
	Low wDE + atropine	190.3 ± 9.0§	1.2 ± 0.4	1.1 ± 0.1§	0.3 ± 0.1
	Air + atropine	241.0 ± 22.6	6.7 ± 2.9	7.7 ± 3.8	4.5 ± 1.9

SDNN - standard deviation of NN intervals
RMSSD - square root of the mean squared difference of successive NNs
LF/HF – low frequency/high frequency
HC030031 – TRPA1 antagonist
RR – ruthenium red, TRP antagonist
SB366791 – TRPV1 antagonist

*significantly different from Air controls, $p < 0.05$
§ significantly different from Low wDE, $p < 0.05$

III. Figures

A. Supplemental Material, Figure 1.

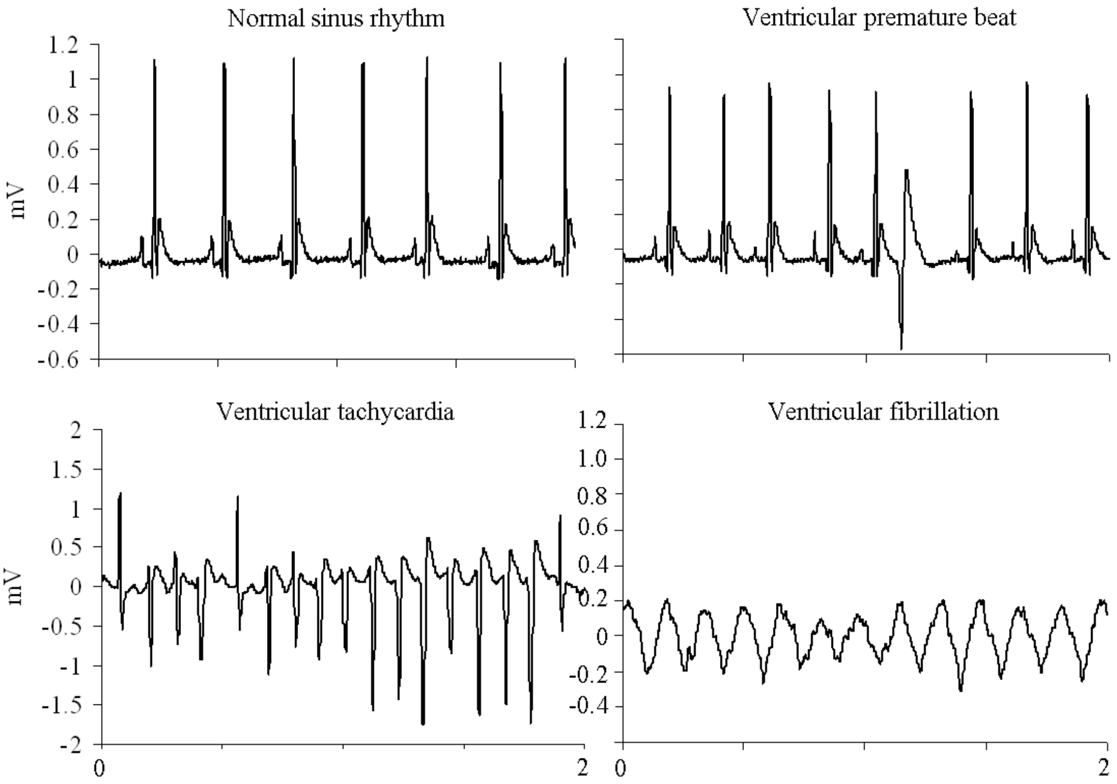

Supplemental Material, Figure1. Typical electrocardiogram (ECG) traces showing normal sinus rhythm, ventricular premature beat (VPB), ventricular tachycardia (VT), and ventricular fibrillation (VF). Given our previous ECG characterization of VF based on the steep drop in blood pressure (Hazari et al. 2009), for the current study, VF was marked as a random (variable amplitude and frequency) ECG waveform with no clear P-wave or QRS complex, and a wandering baseline.

B. Supplemental Material, Figure 2.

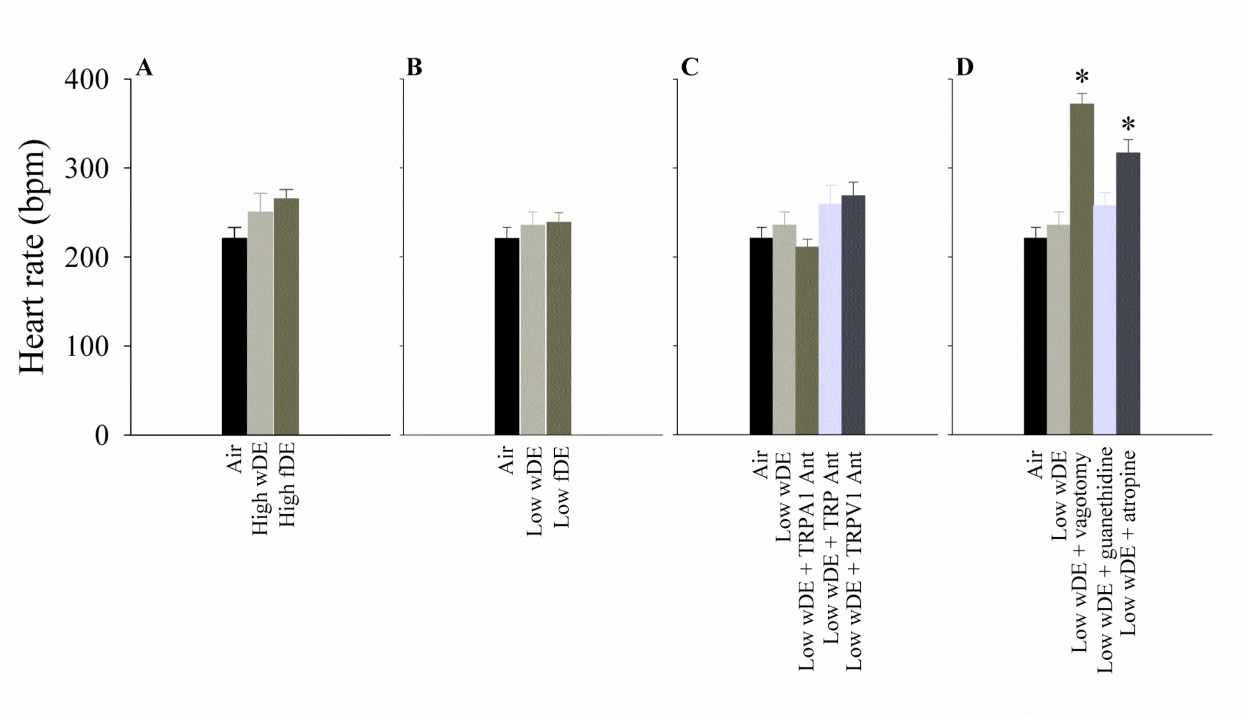

Supplemental Material, Figure 2. Diesel exhaust mediated increase in heart rate is only prevented by blocking TRPA1. One day following exposure, SH rats exposed to high wDE or fDE (**A.**), or low wDE or fDE (**B.**) had slightly higher HR when compared to controls. Pre-treatment with the TRPA1 antagonist prevented the increase in HR due to low wDE, but the TRP antagonist and TRPV1 antagonist caused HR to become further increased (**C.**). Guanethidine treatment of low wDE-exposed rats caused a slight increase, and vagotomy or atropine caused a significant increase in HR (**D.**). Values are mean ± SEM; * significantly different from controls; $p < 0.05$, n = 5-6.

C. Supplemental Material, Figure 3.

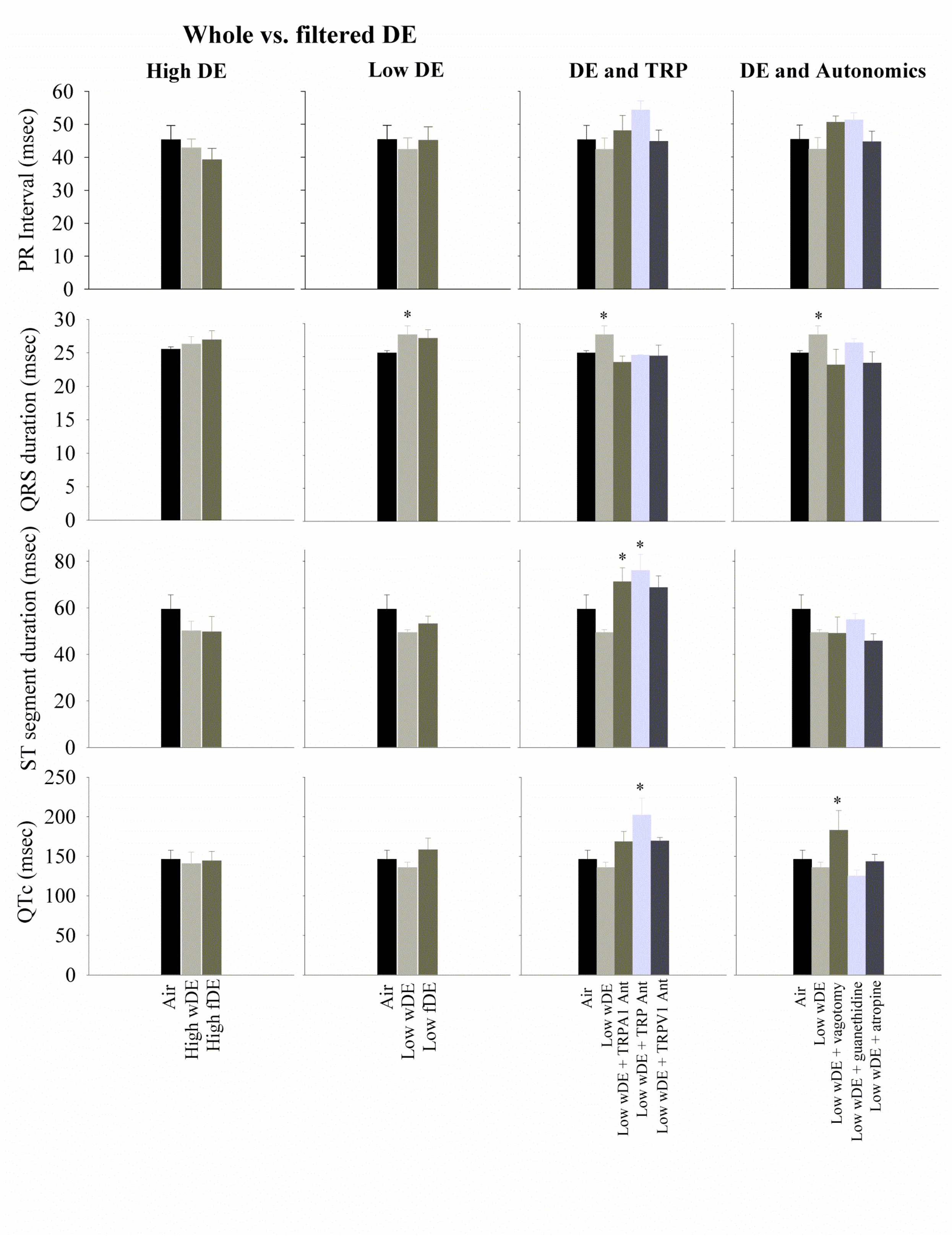

Supplemental Material, Figure 3. Exposure to diesel exhaust causes a prolongation of ventricular depolarization and shortening of repolarization in the ECG. **High DE and Low DE** - SH rats exposed to high wDE or fDE, or low wDE or fDE, had an increase in QRS duration and decrease in ST segment duration when compared to air-exposed controls. **DE and TRP** - Treatment of rats with the TRPA1, TRP or TRPV1 antagonist before low wDE prevented the increase in QRS duration, and decrease in ST segment duration. **DE and Autonomics** – Vagotomy and atropine only prevented the increase in QRS duration caused by low wDE. Guanethidine treatment only partially reversed the decrease in ST segment duration caused by low wDE. Values are mean ± SEM; * significantly different from controls; $p < 0.05$, $n = 5-6$.

IV. References

Hazari MS, Haykal-Coates N, Winsett DW, Costa DL and Farraj AK. 2009. A single exposure to particulate or gaseous air pollution increases the risk of aconitine-induced cardiac arrhythmia in hypertensive rats. Tox. Sci. 112(2):532-542.

Walker MJ, Curtis MJ, Hearse DJ, Campbell RW, Janse MJ, Yellon DM, et al. 1988. The Lambeth Conventions: guidelines for the study of arrhythmias in ischaemia, infarction, and reperfusion. Cardiovasc. Res. 22:447-455.
